# Concordance Between Clinical and Laboratory Diagnosis of Abnormal Vaginal Discharge in Chilean Women

**DOI:** 10.1055/s-0041-1735299

**Published:** 2021-09-21

**Authors:** Angélica Melo, Ximena Ossa, Giselle Fetis, Lorena Lazo, Luis Bustos, Flery Fonseca-Salamanca

**Affiliations:** 1 Pathological Anatomy Department.; 2 Public Health Department and CIGES.; 3 Obstetrics and Gynecology Department.; 4 Preclinical Sciences Department.; 5 Molecular Immunoparasitology Laboratory, Center of Excellence in Translational Medicine, Scientific-Technological Nucleus in Bio-resources. Faculty of Medicine. Universidad de La Frontera, Temuco,Chile.

**Keywords:** vaginal discharge, laboratory diagnosis, bacterial vaginosis, candidiasis, *trichomonas vaginalis*, corrimento vaginal, diagnóstico laboratorial, vaginose bacteriana, candidíase, trichomonas vaginalis

## Abstract

**Objective** To determine the concordance between the clinical diagnosis of women with abnormal vaginal discharge (AVD) and laboratory results using molecular detection and observation of the vaginal microbiota.

**Methods** Cross-sectional study conducted in 2018 in Temuco, Chile. A total of 25 midwives from 12 health centers participated. A total of 125 women > 18 years old, volunteers, were recruited. The sample of the posterior vaginal fornix was obtained by speculoscopy. Characteristics of the discharge and of the external and internal genitalia were observed. Gram staining was used to observe vaginal microbiota, blastoconidia and pseudohyphae, and polymerase chain reaction was used for the detection of *Trichomonas vaginalis* and *Candida albicans* . The Cohen kappa coefficient was used in the concordance analysis.

**Results** Out of a total of 125 women with AVD, 85.6% consulted spontaneously and 14.4% were diagnosed clinically during a routine check-up. Absolute concordance was significant ( *p* = 0.0012), with an agreement of 13.6%. The relative concordance was significant, but fair for bacterial vaginosis (Kappa = 0.21; *p* = 0.003) and candidiasis (Kappa = 0.22; *p* = 0.001), and slight for trichomoniasis (Kappa = 0.14; *p* = 0.009). The percentage of coincidence of the diagnoses (single or mixed) by laboratory and midwives was: bacterial vaginosis 63.2% (12/19), candidiasis 36.5% (27/74), and trichomoniasis 12.5% (4/32). There was 20% coinfection. A total of 36% of the clinical diagnoses of AVD had negative laboratory tests.

**Conclusion** The vulvovaginitis conditions candidiasis and trichomoniasis appear to be overdiagnosed, and bacterial vaginosis appears to be underdiagnosed by the clinical diagnosis when compared with the laboratory diagnosis. The low concordance obtained shows the importance of complementing the clinical diagnosis with a laboratory study of AVD, particularly in women with failed treatments and/or coinfections with unspecific and varying signs and symptoms.

## Resumo

**Objetivo** Determinar a concordância entre o diagnóstico clínico de mulheres com corrimento vaginal anormal (AVD) e os resultados laboratoriais por meio da detecção molecular e observação da microbiota vaginal.

**Métodos** Estudo transversal realizado em 2018 em Temuco, Chile. Participaram 25 parteiras de 12 centros de saúde. Um total de 125 mulheres > 18 anos, voluntárias, foram recrutadas. A amostra do fórnice vaginal posterior foi obtida por especuloscopia. Foram observadas características da secreção e da genitália externa e interna. A coloração de Gram foi usada para observar a microbiota vaginal, blastoconídios e pseudo-hifas, e a reação em cadeia da polimerase foi usada para a detecção de *Trichomonas vaginalis* e *Candida albicans* . O coeficiente kappa de Cohen foi usado na análise de concordância.

**Resultados** De um total de 125 mulheres com AVD, 85,6% consultaram espontaneamente e 14,4% foram diagnosticados clinicamente durante um check-up de rotina. A concordância absoluta foi significativa (p = 0,0012), com concordância de 13,6%. A concordância relativa foi significativa, mas razoável para vaginose bacteriana (Kappa = 0,21; p = 0,003) e candidíase (Kappa = 0,22; p = 0,001), e leve para tricomoníase (Kappa = 0,14; p = 0,009). O percentual de coincidência dos diagnósticos (solteiros ou mistos) por laboratório e parteiras foi: vaginose bacteriana 63,2% (12/19), candidíase 36,5% (27/74) e tricomoníase 12,5% (4/32). Houve 20% de coinfecção. Um total de 36% dos diagnósticos clínicos de AVD tiveram exames laboratoriais negativos.

**Conclusão** As condições de vulvovaginite candidíase e tricomoníase parecem ser sobrediagnosticadas, e a vaginose bacteriana parece ser subdiagnosticada pelo diagnóstico clínico quando comparado com o diagnóstico laboratorial. A baixa concordância obtida mostra a importância de complementar o diagnóstico clínico com estudo laboratorial de AVD, principalmente em mulheres com falha de tratamento e / ou coinfecções com sinais e sintomas inespecíficos e variáveis.

## Introduction

In Chile, consultation with a midwife is the entry into the Sexual and Reproductive Health Program of the Ministry of Health. 1 In primary health care, the midwife is the one who treats the symptomatic woman consulting for abnormal vaginal discharge (AVD), as well as when the finding is made during a routine prenatal or gynecological check-up. The midwife makes a visual and clinical diagnosis (World Health Organization [WHO] syndromic approach) 2 and treats this pathology with no more aid than a suitable semiological process. Only in very special cases are the samples of vaginal discharge sent to the laboratory or is the woman referred to a specialist (gynecologist), when the clinical examination suggests a more complex case.

The frequency of vulvovaginitis in Chilean adolescents and young adults is of ∼ 12.8%, 3 and, when including older women, it reaches ∼ 46.5%, 4 which implies a significant number of consultations in primary health centers, considering that ∼ 80% of the population is registered in the public health system. 5

Abnormal vaginal discharge includes a heterogenous group of vaginal secretions of diverse etiology constituting, in addition to a frequent reason for consultation, a complex public health issue, because they cause a deterioration in the quality of life of women. 6 7 Abnormal vaginal discharge could indicate an altered state in the vaginal microbiota, causing an infection in the reproductive tract, which can increase the frequency of consultation, as well as the predisposition to other sexually transmitted infections and to more severe conditions such as pelvic inflammatory disease, infertility, premature birth, and neonatal infection, among others. 8 9 10

The most frequent etiology of AVD is of infectious origin, in which parasites, fungi, and bacteria can be involved. The main clinical diagnoses of vaginitis or vulvovaginitis point to trichomoniasis, candidiasis, and bacterial vaginosis (BV). 4 11 The signs and symptoms in these infections are often uncharacteristic because they are not always caused by a single microorganism; there can be two or more, making it difficult for the midwife to make an accurate diagnosis and sowing doubts regarding the effectiveness of the treatment indicated. There is evidence that the correlation between the clinical and laboratory diagnosis is poor 12 13 ; however, in many countries such as Chile, the use of making a clinical diagnosis (based on signs and symptoms) without a laboratory diagnosis before indicating treatment for an AVD continues.

The most used laboratory diagnostic methods are nucleic acid amplification tests, which can identify sequences of microorganisms with great specificity and sensitivity, and observation of the vaginal microbiota in vaginal discharge smears, applying a standardized reading, such as the Nugent method. 14

Chilean primary health centers do not have their own laboratories, so when a more specific analysis is required, the samples must be sent to a centralized laboratory that does not have the resources necessary to cope with a high demand. This forces midwives to sharpen their clinical skills and to make as objective a syndromic diagnosis as possible; nevertheless, there is a shortage of concordance studies in Chile to enable knowledge of how much their clinical diagnosis, and consequently the treatment, fits with or approaches reality.

The objective of the present study was to determine the concordance between the clinical diagnosis of women with AVD made by midwives in public health centers in Chile and the laboratory diagnosis by means of molecular detection and microscopic observation of the state of the vaginal microbiota.

## Methods

### Study Design

Cross-sectional study.

### Setting

The present study was conducted in primary health centers in Temuco, Chile, in 2018. This city is among the 5 most populated in the country and is located in its poorest region, in the central-southern zone, with a high proportion of the population served by the public health system. 5 15

### Characteristics of the Sample

Midwives working in primary care centers who agreed to participate in the present study and invited women > 18 years old who had consulted spontaneously for AVD or had AVD diagnosed clinically during a routine check-up to participate. All women voluntarily agreed to participate in the present study. The exclusion criteria were: sexual intercourse in the last 48 hours, cognitive deterioration, menstruation or any bleeding, antimicrobial treatment in the last 30 days, immunosuppression or treatment with immunosuppressants.

### Data Collection

An anonymous survey was applied (using a code), previously tested and standardized, with which the sociodemographic and clinical data of the women (signs observed by the midwife) were successfully obtained. Clinical data were obtained through clinical examination including speculoscopy.

### Vaginal Discharge Sample Collection

Using a speculum, 2 samples were taken from the posterior vaginal fornix with a sterile cotton swab, making 1 smear for each sample, which were then transferred to the laboratory in 1X phosphate buffered saline within 24 hours. The external and internal genitalia, as well as the characteristics of the discharge, were observed.

#### Gram-Stained Vaginal Discharge Smears

In the laboratory, vaginal microbiota was classified by applying the Nugent criteria: normal (NM) with a score from 0 to 3; intermediate (IM) from 4 to 6; and microbiota with BV from 7 to 10 points. 16 Bacterial vaginosis and intermediate microbiota were included to establish the concordance with the clinical diagnosis of BV and nonspecific AVD. The presence of *Trichomonas vaginalis* , blastoconidia, and pseudohyphae was also recorded.

#### DNA Extraction

The DNA was isolated with the E.Z.N.A. Tissue DNA kit (Omega Bio-Tek, Norcross, Georgia, USA) and was stored at - 80°C until processing. As an internal control, polymerase chain reaction (PCR) was used for the β-globin gene, using the primers GH20/PCO4. The positive controls were: genomic DNA, clinical samples positive for *T. vaginalis* and *Candida albicans* by culture.

#### Detection of the Microorganisms

Conventional simple PCR was used for *T. vaginalis* and *C. albicans* , with primers previously published. 17 18 In the concordance analysis, all the samples, *C. albicans* and non *-albicans Candida* , were named as Candida spp.

### Variables

Characteristics of the external genitalia: healthy, presence of erythema, edema, excoriations or other (could record more than one). Characteristics of the internal genitalia: vaginal erythema, cervix erythema, “strawberry cervix,” cervix fissuring or other (could record more than one). Characteristics of the vaginal discharge: quantity (determined by the amount of filling of the lower valve of the speculum as: mild, only leaflet edge secretion; moderate, fill half the leaflet concavity; and sever, fill the leaflet concavity completely), color (determined by a palette of 15 colors), appearance (determined subjectively by observation of the aspects: thick, curdy-white, thin, frothy, adherent or other) and description of the odor (determined subjectively by smell). Clinical diagnosis: midwives do not have other tools like measurement of vaginal pH and the performance of amine tests for clinical diagnosis, only clinical syndromic approach for diagnosis of trichomoniasis, candidiasis, bacterial vaginosis, nonspecific AVD (when the clinical characteristics were unspecific) or other. One or more diagnoses could be recorded. Laboratory results were annotated similarly, and, later, new diagnostic variables that incorporated the combination patterns in the cases of mixed infections were constructed.

### Statistical Analysis

A descriptive analysis of patient characteristics was performed. Concordance tables were implemented to establish agreement between the clinical and laboratory diagnosis according to frequency and to a concordance analysis using the Cohen kappa coefficient ( *p* < 0.05). 19 The nomenclature was standardized to perform this analysis: absolute concordance defined when the clinical diagnoses, either single or mixed, coincided fully with the laboratory diagnosis; relative concordance defined when one of the clinical diagnoses, regardless of it being single or mixed, agreed with one of the laboratory results, regardless if a single infection or a coinfection was detected. A frequency analysis of clinical signs was added according to the etiological agent found in the laboratory.

### Ethical Aspects

The Scientific Ethics Committee of the Universidad de La Frontera (Folio: 017/17, Act N°045/17 CEC-UFRO) and the Scientific Ethics Committee of the South Araucanía Health Service (resolution through letter dated November 28, 2017) authorized the present study. Signed informed consent was required. All women were treated with the routine protocol used for pathological vaginal discharge.

## Results

A total of 25 midwives from 12 primary health centers participated, and they recruited 125 women with AVD, of which 85.6% consulted spontaneously and 14.4% were diagnosed clinically during a routine check-up. The women were between 18 and 61 years old; 85.0% resided in urban areas, 60.8% had elementary or secondary education, 39.2% had technical or university education, 65.6% had a partner (includes married participants and those who had a partner, regardless of whether they lived together or not); and 59.2% used a contraceptive method (66.2% used hormonal contraceptives, 21.6% used condoms, and 12.2% used intrauterine devices).

The absolute concordance was significant ( *p* = 0.0012) but slight (Kappa = 0.07), with an exact agreement of 13.6%. The relative concordance for each of the diagnoses was significant but fair for BV (Kappa = 0.21; *p* = 0.003) and candidiasis (Kappa = 0.22; *p* = 0.001), and slight for trichomoniasis (Kappa = 0.14; *p* = 0.009). 19

Global single clinical diagnoses were 72.0% (90/125), and mixed clinical diagnoses were 28.0% (35/125). The percentage of coincidence of the diagnoses (single or mixed) by laboratory and midwives was: bacterial vaginosis 63.2% (12/19), candidiasis 36.5% (27/74), and trichomoniasis 12.5% (4/32).

Applying the Nugent criteria in the reading of the Gram ( *n* = 125), it was observed: 44.0% of cases with normal microbiota, 19.2% with intermediate microbiota, 35.2% with BV, 0.8% with aerobic vaginitis, and 0.8% without bacterial microflora. Among the 24 cases identified with intermediate microbiota, 11 presented coinfection with *T. vaginalis* or *Candida* spp. In the Gram, 18.4% of cases with pseudohyphae and/or blastoconidia and 2.4% with *T. vaginalis* were observed. Using specific PCR, 19.2% of the cases were detected with *C. albicans* , and 4.8% with *T. vaginalis* .

Although *Chlamydia trachomatis* (CT) infection was not included in the concordance analysis, it was investigated in the laboratory by PCR, detecting an estimated frequency of 14.9% in this group of women.

Table 1 shows the distribution of clinical diagnoses according to the laboratory diagnoses. The percentage of negative cases observed in the laboratory in relation to the clinical diagnoses of candidiasis and nonspecific ADL is highlighted. A total of 20.0% (25/125) of the women had a coinfection: Candida spp./IM, Candida spp./BV, *T. vaginalis* /IM, *T. vaginalis* /BV, *T. vaginalis* /Candida spp./BV, and *T. vaginalis* /Candida spp./IM.

**Table 1:** Clinical diagnosis of women with abnormal vaginal discharge *versus* laboratory diagnosis ( *n* = 125)

Unique clinical diagnosis a	*n* (%)	d Laboratory diagnosis *n* (%)						
		CAN(+)/MN	CAN(+)/MI	CAN(+)/VB	IM*	TV(+)/Other b	BV	NM/TV(-)/CAN(-)
CAN	53 (42.4)	10 (18.9)	2 (3.8)	5 (9.4)	5 (9.4)	1 (1.9)	6 (11.3)	24 (45.3)
nAVD	21 (16.8)	0	0	2 (9.5)	4 (19.0)	0	6 (28.6)	9 (42.8)
TV	10 (8.0)	0	0	0	2 (20.0)	3 (30.0)	4 (40.0)	1 (10.0)
BV	6 (4.8)	0	1 (16.7)	1 (16.7)	0	1 (16.7)	2 (33.3)	1 (16.7)
Mixed diagnosis								
CAN-TV	10 (8.0)	2 (20.0)	4 (40.0)	0	0	0	2 (20.0)	2 (20.0)
BV-nAVD	6 (4.8)	0	0	1 (16.7)	0	0	3 (50.0)	2 (33.3)
CAN- nAVD	5 (4.0)	0	1 (20.0)	1 (20.0)	1 (20.0)	0	1 (20.0)	1 (20.0)
TV- nAVD	5 (4.0)	0	0	0	0	0	2 (40.0)	3 (60.0)
Other combinations c	9 (7.2)	0	0	1 (11.1)	1 (11.1)	1 (11.1)	4 (44.5)	2 (22.2)
TOTAL	125	12 (9.6)	8 (6.4)	11 (8.8)	13 (10.4)	6 (4.8)	30 (24.0)	45 (36.0)

a Clinical Diagnosis: CAN: Candidiasis, TV: Trichomoniasis, BV: Bacterial vaginosis, nAVD: nonspecific abnormal vaginal discharge.

b TV(+)/Other (clinical diagnosis): TV(+)/CAN(+)/IM (1 TV),TV(+)/CAN(+)/BV (1 CAN), TV(+)/IM (1 TV, 1 VB), TV(+)/BV (1TV, 1TV-CAN-BV).

c Other combinations of clinical diagnoses (laboratory diagnosis): TV-BV (1 NM/TV(-)/CAN(-), 2 BV), CAN-TV (1 NM/TV(-)/CAN(-), 1 BV), TV-CAN- nAVD (1 CAN(+)/BV, 1 IM),TV-CAN-BV (1 TV(+)/Other, 1 BV).

d Laboratory Diagnosis: TV: *Trichomonas vaginalis* , CAN: *Candida* spp. Microorganisms identified by PCR and/or presence of blastoconidia or pseudohyphae in Gram staining. Reading of the vaginal microbiota according to Nugent criteria: NM: normal microbiota, IM*: intermediate microbiota (only cases that presented as unique this laboratory diagnosis alteration were considered), BV: Bacterial vaginosis, NM/TV(-)/CAN(-): Negative cases.

Table 2 shows the characteristics of the vaginal discharge observed by the professionals when the samples were taken. Three cases with no record of discharge quantity were excluded from the analysis ( *n* = 122). A moderate quantity of vaginal discharge was the most frequent observed in women both negative and positive for BV and negative cases. The appearance of the discharge as thick/curdy-white, thick, curdy-white, and thin were the most frequent. It is worth noting the thick, curdy-white and thick/curdy-white appearance in TV(-)/CAN(-)/NM cases, and the thin and thin/frothy appearance in BV cases.

**Table 2:** Characteristics of abnormal vaginal discharge of women according to laboratory diagnosis ( *n* = 125)

Characteristics of vaginal discharge		*n* (%)	c Laboratory diagnosis *n* (%)						
			CAN(+)/NM	CAN(+)/IM	CAN(+)/BV	IM*	TV(+)/Other d	BV	NM/TV(-)/CAN(-)
a Quantity	mild	28 (23.0)	2 (7.1)	0	2 (7.1)	4 (14.3)	0	8 (28.6)	12 (42.9)
	moderate	70 (57.4)	8 (11.4)	6 (8.6)	6 (8.6)	5 (7.1)	2 (2.8)	17 (24.3)	26 (37.1)
	severe	24 (19.7)	2 (8.3)	2 (8.3)	3 (12.5)	4 (16.7)	4 (16.7)	3 (12.5)	6 (25.0)
Appearance	thick / curdy-white	25 (20.0)	6 (24.0)	2 (8.0)	3 (12.0)	2 (8.0)	1 (4.0)	2 (8.0)	9 (36.0)
	thick	22 (17.6)	1 (4.5)	1 (4.5)	1 (4.5)	2 (9.1)	1 (4.5)	6 (27.3)	10 (45.5)
	curdy-white	20 (16.0)	3 (15.0)	2 (10.0)	3 (15.0)	2 (10.0)	0	1 (5.0)	9 (45.0)
	thin	18 (14.4)	1 (5.6)	1 (5.6)	1 (5.6)	2 (11.1)	0	6 (33.3)	7 (38.9)
	thin /frothy	10 (8.0)	0	0	0	1 (10.0)	2 (20.0)	7 (70.0)	0
	thick /adherent	8 (6.4)	0	0	0	1 (7.7)	0	4 (38.8)	3 (38.5)
	frothy	5 (4.0)	0	0	1 (20.0)	1 (20.0)	0	2 (40.0)	1 (20.0)
	Other combinations b	17 (13.6)	1 (10.0)	2 (20.0)	2 (10.0)	2 (10.0)	2 (10.0)	2 (10.0)	6 (40.0)

a Quantity: *n* = 122(3 cases that had no history), the quantity of vaginal discharge is referred to how much volume remains in the concavity of the leaflet when the speculum is removed.

b Other combinations of the appearance (Laboratory diagnosis). With one case: adherent (CAN(+)/BV), thick/frothy (BV), thin/curdy-white(NM/TV(-)/CAN(-)),thin/frothy/adherent (BV), thin/curdy-white/adherent (NM/TV(-)/CAN(-)), thick/frothy/adherent (TV/Other), thin/adherent (IM). With 2 cases: thin/thick (TV (+)/other, NM/TV(-)/CAN(-)), curdy-white/frothy (CAN(+)/NM, CAN(+)/IM). With 3 cases: curdy-white/adherent (CAN (+)/BV, IM, NM/TV(-)/CAN(-)), thick/curdy-white/adherent (2 NM/TV(-)/CAN(-), CAN(+)/IM).

c Laboratory Diagnosis: TV: *Trichomonas vaginalis* , CAN: *Candida* spp. Microorganisms identified by PCR and/or presence of blastoconidia or pseudohyphae in Gram staining. Reading of the vaginal microbiota according to Nugent criteria: NM: normal microbiota, IM*: intermediate microbiota (only cases that presented as unique this alteration were considered), BV: Bacterial vaginosis. NM/TV(-)/CAN (-): negative cases.

d TV(+)/Other(clinical diagnosis): TV(+)/CAN(+)/IM (1 severe vaginal discharge, 1 thin /frothy appearance), TV(+)/CAN(+)/BV(1 severe vaginal discharge, 1 thick / curdy-white appearance), TV(+)/IM (1 severe, 1 moderate vaginal discharge, 1 thick and 1 thin /frothy appearance), TV(+)/BV (1 severe and the other moderate vaginal discharge case).

In relation to the color of the discharge, the most frequent were: white 41.6% (52/125), yellowish-white 20.8% (26/125), grayish-white 15.2% (19/125), and pale yellow 6.4% (8/125). Regarding the infection and the color: white/grayish-white *Candida* spp./NM 83.3% (10/12), intermediate microbiota 46.2% (6/13), BV 46.7% (14/30), and pale yellow *T. vaginalis* (considering all the cases) 50.0% (3/6). The odor of the discharge when the sample was taken was not included in the analysis, since there were few criteria defined by the midwives for its evaluation, and the description of this variable varied widely. Regarding signs and symptoms (there were 2 cases with no records): 39.8% (49/123) of the women were asymptomatic, 8.1% (10/123) presented signs only in the external genitalia, 30.9% (38/123) in the internal genitalia, and 21.1% (26/123) in both. Table 3 shows the clinical signs that the women presented when the vaginal sample was taken. Bacterial vaginosis had the highest number of cases without signs in external genitalia (76.6%) and internal genitalia (46.4%). In internal genitalia, the data were analyzed with *n* = 123, and it was observed that in those presenting no signs (59/123), 55.9% were diagnosed with some of the infections tested in the present study.

**Table 3:** Characteristics of the genitals of women with clinical diagnosis of abnormal vaginal discharge ( *n* = 125)

Genitalia	c Characteristics	*n* (%)	b Laboratory diagnosis *n* (%)						
			CAN(+)/NM	CAN(+)/IM	CAN(+)/BV	IM	TV(+)/Other	BV	NM/TV(-)/CAN(-)
External	no signs	90 (72.0)	8(8.9)	2 (2.2)	8 (8.9)	11 (12.2)	1 (1.1)	23 (25.6)	37 (41.1)
	erythema	25 (20.0)	1(4.0)	4 (16.0)	3 (12.0)	2 (8.0)	4 (16.0)	5 (20.0)	6 (24.0)
	eryth/escoriation	5 (4.0)	3(60.0)	1 (20.0)	0	0	1 (20.0)	0	0
	Others external	5 (4.0)	0	1 (20.0)	0	0	0	2 (40.0)	2 (40.0)
a Internal	no signs	59 (48.0)	9(15.3)	0	3 (5.1)	7 (11.9)	1 (1.7)	13 (22.0)	26 (44.0)
	vageryth	181 (4.6)	1(5.5)	1 (5.5)	1 (5.5)	3 (16.7)	2 (11.0)	6 (33.3)	4 (22.2)
	vageryth/cxeryth	11 (8.9)	1(9.0)	2 (18.2)	4 (36.4)	0	1 (9.0)	1 (9.0)	2 (18.2)
	cxeryth	8 (6.5)	0	1 (12.5)	0	3 (37.5)	0	0	4 (50.0)
	vageryth/cxeryth/cxfiss	6 (4.9)	0	2 (33.3)	0	0	2 (33.2)	1 (16.6)	1 (16.6)
	cervix fissuring	5 (4.0)	1(20.0)	0	0	0	0	2 (40.0)	2 (40.0)
	cxeryth/strawcx	5 (4.0)	0	2 (40.0)	1 (20.0)	0	0	1 (20.0)	1 (20.0)
	Others internal	11 (8.9)	0	0	2 (18.2)	0	0	4 (36.4)	5 (45.4)

a Internal: *n* = 123(2 cases that had no history).

b Laboratory Diagnosis: TV: *Trichomonas vaginalis* , CAN: *Candida* spp. Microorganisms identified by PCR and/or presence of blastoconidia or pseudohyphae in Gram staining. Reading of the vaginal microbiota according to the Nugent criteria: NM: normal microbiota, IM*: intermediate microbiota (only cases that presented as unique this laboratory diagnosis alteration, were considered), BV: Bacterial vaginosis. NM/TV(-)/CAN(-): Negative cases.

c Characteristics: eryth: erythema, strawcx: “Strawberry” cervix, cxfiss: cervix fissuring, cx: cervical or cervix, vag: vaginal, exc:excoriation, vageryth:vaginal erythema, cxeryth: cervix erythema, vageryth/cxeryth/reddotted: vaginal erythema plus cervical erythema plus strawcx, vageryth/erithcx/strawcx /cxfiss: vaginal erythema plus cervical erythema plus “strawberry cervix” plus cervix fissuring.

d TV(+)/Other(genitalia characteristics). External: TV(+)/CAN(+)/IM (1 erythema), TV(+)/CAN(+)/BV (1 no signs), TV(+)/IM (2 erythema), TV(+)/BV (1 erythema, 1 eryth/escoriation). Internal: TV(+)/CAN(+)/IM (1 vageryth), TV(+)/CAN(+)/BV (1 no signs), TV(+)/IM (1 vageryth/cxeryth, 1 vageryth/cxeryth/cxfiss), TV(+)/BV (1 vageryth, 1 vageryth/cxeryth/cxfiss). Other external (laboratory diagnosis): escoriation (2 BV), edema (2 NM/TV(-)/CAN(-)), erythema/edema(1 CAN(+)/IM). Other internal (laboratory diagnosis): vageryth/cxfiss(1 CAN(+)/BV, 2 BV), cxeryth/cxfiss (2 NM/TV(-)/CAN(-), 1 BV), cxeryth/strawcx /cxfiss (1 NM/TV(-)/CAN(-), 1 CAN(+)/BV), vageryth/cxeryth /strawcx/cxfiss (1 NM/TV(-)/CAN(-), 1 BV), vageryth/strawcx (1 NM/TV(-)/CAN(-)).

## Discussion

Abnormal vaginal discharges (AVDs) from women attending public health centers were analyzed to evaluate the concordance between the clinical diagnosis and the laboratory diagnosis.

The absolute concordance was 13% between the syndromic diagnosis and that of the laboratory, which was considered significant but poor. In this sense, Barry et al. 13 reported a higher kappa value (< 0.20) than the one obtained in the present study, although the concordance was equally slight or poor. Low concordance has also been found in other studies, such as in the one by Chauhan et al., 20 who determined positive results in ∼ 35% of the clinically positive samples, and in the one by Sonkar et al., 21 who demonstrated 17% of concordance between the clinical and the laboratory diagnoses.

Regarding the percentage of coincidence of the diagnoses (laboratory and midwives), Tellapragada et al. 22 report similar values for BV and candidiasis, but not for trichomoniasis, which was higher (37.6 versus 12.5% found in the present study).

Over a third of the participants (36.0%) were negative for the laboratory examination even though the women presented symptoms or signs compatible with an AVD, diagnosed clinically. However, it cannot be ruled out that these women may have been positive for other vaginal infections that were not tested in the present study. A physiological discharge can present some abnormal-looking features, such as thick and sticky, which have been described during the menstrual cycle, with the use of contraceptives, or with sexual stimulation. 23 In the women in the present study who had negative results in the laboratory, 75% used hormonal contraceptives and 6.3% used intrauterine devices, which can explain, in part, the complexity in clinically defining AVD. Without a doubt, the measurement of the pH, the amine test, and a microscopy of the vaginal fluid at the time of the gynecological consultation can help to clarify the type of discharge. However, in our setting, at the primary health care level, these tests are not performed.

In the present study, a great variability can also be noted in the clinical parameters midwives use for the diagnosis, but there are also significant differences in the clinical manifestation of the different vaginal infections. Generally, the color, quantity and appearance of the discharge did not appear homogenously for a particular infection. A moderate quantity of discharge was the most frequent, observed in women with *Candida* spp., BV and negatives. Paladine et al. 24 describe the discharge of candidiasis as white/thick/curdy-white; however, in our study, the thick/curdy-white appearance was noted cross-sectionally from negative samples in the laboratory up to BV, intermediate microbiota, and *T. vaginalis* , although with a slightly greater proportion in the cases with *Candida* spp./normal microbiota.

Presently, with a more heterogenous urban population, a higher variability in sexual habits (multiple sexual partners in a short time), the appearance of new infections, such as *Mycoplasma genitalium* or *Mycoplasma hominis* , 25 26 27 or the presence of a dysbiotic microbiota (BV, intermediate microbiota), the presentation of the discharge and of the signs and symptoms can be very similar to that of infections considered classic, such as candidiasis and trichomoniasis.

In the present study the most frequent syndromic diagnosis was candidiasis (42.4%), and BV was the least frequent (4.8%); however, as a global laboratory diagnosis, BV was the most frequent (35.2%) and *T. vaginalis* the least frequent (4.8%). The low estimated frequency of *T. vaginalis* in the present study could be useful knowledge, leading the treating professional to rethink that the woman could present a BV, since this shares characteristics of the discharge (as thin/frothy) with *T. vaginalis* more than with candidiasis. 9 On the other hand, cases with *T. vaginalis* were more associated with intermediate microbiota and BV (8.8%) than with *Candida* spp. (6.6%). Das et al. observed something similar, finding that *T. vaginalis* cases increased as the Nugent score increased. 17

Regarding women with candidiasis, the presence of non- *albicans Candida* species in the present study (6 samples) raises the need to genotype these cases, because they could be playing a role in women who experience a recurrence and require a change in treatment. 28

Candidiasis was the most associated syndromic diagnosis in women with CT; however, in the laboratory, it was BV (61.1%). On this aspect, it could be an alert for midwives, since an increased risk of CT has been reported in women with a history of BV. 29

In the external and internal genitalia, erythema was the most frequent clinical sign; nevertheless, it was not characteristic of any specific infection, although it was observed that most cases of BV presented this sign ( Table 2 ). It is remarkable that cases that presented negative results for the examinations performed in the present study also presented erythema. On this point, it could be speculated that some other infection could be present, such as *Mycoplasma genitalium* , 28 or that it could be cytolytic vaginitis 30 31 or vaginal lactobacillosis 32 that could be causing the AVD and the erythema.

Without a doubt, the range of vaginal infections is increasing every day, including not only the “classic” infections, but also others such as *Mycoplasmas* and even enterobacteria that can cause aerobic vaginitis. 33

Finally, a clinical diagnosis considering the signs and symptoms of the woman combined with laboratory techniques (fast and low-cost) incorporated as routine in the health center would help improve the accuracy of a diagnosis. In addition, with a syndromic guide that evaluates each of the aspects in the most objective way possible, a more effective diagnosis could be made, especially for women with failed treatments, mixed infections with unspecific and varying signs and symptoms, or with a history of sexual habits such as multiple partners, use of condoms and/or contraceptive methods.

## Conclusion

Our results show very low concordance between the clinical diagnosis and the laboratory diagnosis, thus demonstrating that a diagnosis based only on clinical signs, symptoms, and characteristics of AVD and not supported by laboratory examinations can lead to a diagnostic error and, therefore, to an inadequate treatment. These findings highlight the importance and the need to incorporate laboratory diagnosis for women with AVD to identify the causal agent prior to treatment.
